# Human Biomonitoring of Glyphosate Exposures: State-of-the-Art and Future Research Challenges

**DOI:** 10.3390/toxics8030060

**Published:** 2020-08-18

**Authors:** Alison Connolly, Marie A. Coggins, Holger M. Koch

**Affiliations:** 1 Institute for Prevention and Occupational Medicine of the German Social Accident Insurance—Institute of the Ruhr-University Bochum (IPA), Bürkle-de-la-Camp-Platz 1, 44789 Bochum, Germany; 2 Centre for Climate and Air Pollution Studies, School of Physics and the Ryan Institute, National University of Ireland, University Road, H91 CF50 Galway, Ireland; marie.coggins@nuigalway.ie

**Keywords:** glyphosate, aminomethylphosphonic acid, AMPA, urine, human biomonitoring, environmental exposures, toxicokinetics, exposure assessment, risk assessment

## Abstract

Glyphosate continues to attract controversial debate following the International Agency for Research on Cancer carcinogenicity classification in 2015. Despite its ubiquitous presence in our environment, there remains a dearth of data on human exposure to both glyphosate and its main biodegradation product aminomethylphosphonic (AMPA). Herein, we reviewed and compared results from 21 studies that use human biomonitoring (HBM) to measure urinary glyphosate and AMPA. Elucidation of the level and range of exposure was complicated by differences in sampling strategy, analytical methods, and data presentation. Exposure data is required to enable a more robust regulatory risk assessment, and these studies included higher occupational exposures, environmental exposures, and vulnerable groups such as children. There was also considerable uncertainty regarding the absorption and excretion pattern of glyphosate and AMPA in humans. This information is required to back-calculate exposure doses from urinary levels and thus, then compare these levels with health-based guidance values. Back-calculations based on animal-derived excretion rates suggested that there were no health concerns in relation to glyphosate exposure (when compared with EFSA acceptable daily intake (ADI)). However, recent human metabolism data has reported as low as a 1% urinary excretion rate of glyphosate. Human exposures extrapolated from urinary glyphosate concentrations found that upper-bound levels may be much closer to the ADI than previously reported.

## 1. Introduction

Glyphosate (N-(phosphonomethyl)glycine) is a broad-spectrum, post-emerging herbicide, which is highly water-soluble and affects the shikimate metabolic pathway mechanism of plants. It was first synthesized as a herbicide in the 1970s by the US firm Monsanto (later acquired by the German company Bayer in 2018). Glyphosate is the highest volume used herbicide in the world; the active ingredient is in over 750 products. Over the past decade, over 8 million metric tons of glyphosate have been applied worldwide [1,2]. Glyphosate is extensively used in the agricultural sector, including on genetically modified crops but it is also used in horticulture, for both amenity and residential purposes. The main biodegradation product of glyphosate is aminomethylphosphonic acid (AMPA). Both glyphosate and AMPA have been detected as residues on crops and plants [3].

Scientific and public discussions on glyphosate have stepped up considerably since 2015 when the International Agency for Research on Cancer (IARC) of the World Health Organization (WHO) classified the chemical as ‘Group 2A—probably carcinogenic to humans’ [4]. European and US agencies have differed in their classification of glyphosate and consider the chemical to be a non-carcinogenic to human substance [3,5,6].

Although regulatory agencies and authorities are predominantly aligned in their evaluation of glyphosate, there are some ambiguities regarding the possible adverse health effects and the magnitude and range of human exposures in both occupational and environmental settings. Some review studies have concluded that exposure to glyphosate is associated with cancer in humans [7,8]. A study from the Agricultural Health Study (AHS) survey (a follow up from a 2001 study) reported that 83% of 54,251 applicators used glyphosate, but the study found no statistically significant associations between glyphosate use and cancer [9]. There are also concerns regarding non-carcinogenic adverse health effects associated with glyphosate, including potential effects on the endocrine system such as thyroid function [10] on the renal [11,12], respiratory [13,14], and reproductive systems [15,16]. 

In recent years, there has been an increasing amount of literature focusing on the evaluation procedures for regulatory risk assessment of pesticides [17,18,19,20]. Particular discussion points include that chronic pesticide studies are only on the active ingredient (i.e., technical glyphosate) and the requirement for more real-world exposure data on the formulated glyphosate products, due to the combined effects on the toxicity [18]. The requirement for post-market surveillance is also discussed. Currently, pre-market testing is highly focused on the active ingredient and requires just one intended use scenario. This intended use can vary once on the market, for example in the case of glyphosate, it can be used by farmers to spray their fields and eradicate weeds before crops are planted, but in practice, it is also used on genetically modified crops and as a pre-harvest desiccant. Post-market surveillance along with harmonized data collection strategies [21] would be of utmost importance for acquiring information on the distribution of exposure levels including non-routine exposure scenarios (e.g., para-occupational exposures) and for real-world human exposures, which would result in more robust regulatory risk assessments [18].

Gillezeau et al. (2019) highlighted that ‘additional studies are urgently needed to evaluate levels of glyphosate and related metabolites in the general population and workers’ are crucial for improving exposure assessments and thus ensuring a more accurate risk characterization [22]. A paucity of data exists regarding glyphosate exposure levels in potentially exposed (sub)populations in occupational and environmental settings and among different family types, e.g., studies focusing on residential, para-occupational, and environmental exposures. This is especially important for vulnerable populations such as children, who have already been shown to exhibit higher levels of glyphosate body burdens than adults [23,24].

Human biomonitoring (HBM) of exposure is the systematic assessment of the levels of chemicals and their metabolites through the analysis of human samples such as blood, urine, hair, or breast milk. HBM, as a measure of actual internal exposure, is considered an essential tool for comprehensive exposure and risk assessments [25]. HBM can be used to identify new chemicals exposures, inform temporal changes, and the distribution of exposure among groups, especially vulnerable groups (e.g., children). HBM is a method capable of linking potential external exposure (often described or determined in worst-case scenarios) to actual internal concentrations, thus providing reliable exposure information (extend and magnitude) to be linked to toxicological data for risk characterization [26,27]. HBM plays a decisive role in health prevention and risk management, providing information for regulatory agencies and policy-makers [26].

There have been a limited number of HBM studies on glyphosate. Some studies cover occupational exposure levels only or do not include AMPA, glyphosate’s major biodegradation product/metabolite. Moreover, rather surprisingly, for a chemical used for so long and in such quantities, there is considerable ambiguity about the rate of oral absorption of glyphosate, its systemic availability, its metabolism to AMPA, and the urinary excretion fractions of both compounds in humans. Understanding human absorption, metabolism, distribution, and excretion are of general importance for toxicological considerations but is also essential for back-calculating daily intakes of glyphosate from urinary levels (of glyphosate and/or AMPA) and to compare these calculated intakes with effective doses from animal studies (such as the NOAEL) and health-based guidance values for humans such as the acceptable daily intake (ADI) [3] or the reference dose (RfD) [28]. Previous back-calculations from urine concentrations to a daily intake/systemic dose assumed a urinary excretion of 20% of oral glyphosate excreted as unchanged glyphosate in urine [3], with earlier estimates of 30% [29]. Very recent studies suggest that the human’s excretion fractions of glyphosate in urine (as unchanged glyphosate) could be as low as 1% [30,31]. These recent findings directly impact the quantitative significance of urinary glyphosate (and AMPA) as the established exposure biomarker for glyphosate, as well as exposure and risk extrapolations based thereon. While, initially, a 1% urinary excretion might look more favourable than a 20% excretion, the back-calculated oral glyphosate dose would be 20 times higher than previously assumed based on the same urinary glyphosate data.

Thus, the overall objective of this manuscript is to evaluate and standardize the results of the current state-of-the-art in glyphosate exposure assessment using human biomonitoring (urine samples), to evaluate the internal glyphosate concentrations to health-based guidance values, to outline the gaps in knowledge that are still required for interpretation of HBM data (human glyphosate metabolism and excretion), and to propose recommendations for sampling strategies, all of which could inform future studies investigating population exposures to glyphosate.

## 2. Methodology

A critical review of the literature for glyphosate exposure studies using human biomonitoring was conducted by initially researching previous review studies [4,22,32] and then conducting a review of all scientific publications on glyphosate in humans (both occupationally and environmentally exposed populations). Literature searches were performed in Web of Science and PubMed, using the following search terms ‘Glyphosate AND Urine’, ‘Glyphosate AND human’, and ‘Glyphosate AND human biomonitoring’. These search terms were also conducted with the inclusion of ‘AMPA’. There was no language restriction if an English abstract was provided. Publications were excluded if they reported animal studies, environmental matrices (e.g., food residues, air and soil), in vivo and in vitro data. This review excluded studies that were not peer-reviewed publications with the exception of one study [33], but this study was commented on by the German Federal Institute for risk assessment (BfR) [34]. Publications were not reviewed for their quality or ranked on other studies. This resulted in 21 publications investigating glyphosate exposures among humans using a human biomonitoring strategy, specifically, collecting urine samples. Publications were then separated based on whether they were investigating occupational or non-occupational exposures (which included environmental, para-occupational and residential exposures).

The data was extracted from the publications using the following headings: author, year, country of the study, the type of study (e.g., type of workplace), the sampling population, the type of urine sample collected (e.g., spot or full void samples), the analytical method used, and for both glyphosate and AMPA (if analyzed within the study) the limit of detection/quantification (LOD/LOQ), the frequency of detection (% above LOD/LOQ), the average and maximum values.

The studies presented their quantitative urinary concentrations using various approaches, and this study attempted to standardize the results as far as was possible. Thus, in an effort to make comparisons between the studies, results were presented in the most transparent way possible, by standardizing the table for all glyphosate concentrations to micrograms per litre (µg/L) for comparability and reported the average (central tendencies) and the maximum values. The central tendencies were sometimes reported as geometric means, arithmetic means or median values. However, in this study, values were only displayed for studies that had a detection rate of over 50% and if these values referred to the whole population investigated (or a defined sub-population). Several studies evaluated only those samples above the respective LOQ (e.g., derived mean/median concentrations only for this subset). We excluded such reported values from the evaluation in our tables because they are not comparable with each other and strongly depend, among other things, on population composition and the chemical-analytical sensitivities. Due to the small number of studies available, no further statistical analysis was conducted on these studies.

To further evaluate these studies, the results were compared to the appropriate health-based guidance values. In 2015, the European Food Safety Authority (EFSA) published an acceptable daily intake (ADI) value for glyphosate, calculated on the no observed adverse effect level (NOAEL) based on animal studies and by applying a standard uncertainty factor (UF) of 100. The ADI is expressed as the mass of glyphosate, per kilogram of body weight per day (mg/kg b.w./day) [3]. As these are human biomonitoring studies, human toxicokinetic information (e.g., urinary excretion fractions) must be taken into consideration for back-calculation, to extrapolate for external exposures and make it comparable to the EFSA ADI or other guidance values. To demonstrate the influence of the urinary excretion fractions on the external dose, the calculated glyphosate concentrations were compared to the ADI based upon the non-occupationally exposed populations. For best comparability, the same back-calculation approach as Niemann et al. (2015) [32] was used. The concentration of glyphosate in urine is multiplied by the volume of urine (standardized as 2 litres per day) and divided by body weight (standardized to 60 kg) multiplied by urinary excretion fraction of glyphosate 1% and multiplied by EFSA’s ADI (0.5 mg/kg b.w./day) (Equation (1)).

(1) $$\%\;\mathrm{ADI}\;=\frac{{\mathrm{Gly}}_{\mathrm{conc}.}*{\mathrm{Vol}}_{\mathrm{Urine}}}{\mathrm{BW}*F_{\mathrm{ue}}*\mathrm{ADI}}$$

where Gly_conc_ is the concentration of glyphosate measured in urine; Vol_urine_ is standardized as two litres; BW is bodyweight which is standardized at 60 kg; the F_ue_ is the urinary excretion fraction (0.01 F_ue_); and ADI is the acceptable daily intake allowance for glyphosate (0.5 mg/kg b.w./day). 

## 3. Human Biomonitoring Studies on Glyphosate

To date, there is a dearth of peer-reviewed human biomonitoring (HBM) studies internationally that report glyphosate exposures. The majority of HBM exposure studies focus on glyphosate (and only some on its biodegradation product AMPA) in urine samples. There have been studies that report glyphosate concentrations in other biological matrices (e.g., material and umbilical cord serum [35], and breast milk [36]). However, this current article only focuses on studies that report urinary levels. 

Interpretation and comparison of these datasets need to be conducted with caution, as they do not all follow a similar sampling strategy and are focused on different populations, apply various detection/quantification principles (e.g., ELISA or mass spectrometry), and have inherent differences in selectivity/specificity, as well as a wide range of the limit of detection/quantification (LOD/LOQ) ranging from 0.02 to 100 μg/L. Moreover, data presentation and quantitative evaluation within the studies differ considerably. Of the 20 peer-reviewed human biomonitoring studies (plus one non peer-reviewed report) investigating glyphosate exposure via urine sampling, approximately half of the studies (*n* = 10) also analyzed for AMPA, of which only eight found detectable levels.

The sampling strategy for urine sample collection (e.g., spot, first morning, 24-h) and urinary dilution adjustments (creatinine, relative density) have also varied, with the majority of these studies adopting the collection of spot urine samples. Although urine volume and urinary dilution can vary between voids and between subjects [37], only three studies collected 24-h urine samples [38,39,40,41], and one study collected individual full void urine samples noting last time of urination [42]. 

In total, 7 of the 21 studies did not include a urinary dilution adjustment metric; of these seven studies, most of the studies collected spot urine samples. Of the studies that did include a measure of urinary dilution, most measured for creatinine and only a few for relative density, although some of these studies did not report the values. Jayasumana et al. (2015) in their study of agricultural nephropathy reported both unadjusted and creatinine adjusted values, appropriate to the study objectives, as underlying diseases (e.g., kidney disease) could cause fluctuations in creatinine levels [11].

Glyphosate levels found in occupational studies among farmers [24,39,40,43], amenity horticulturists [42,44,45,46], and in pesticide production [47] are summarized in Table 1. The average glyphosate concentrations reported for farmers and horticulturists ranged within rather small boundaries from 1.35 µg/L to 3.2 µg/L, and the maximum values ranged from approximately 10 µg/L to 233 µg/L. A study among farm families (based in South Carolina and Minnesota) reported that 60% of farmers urine samples analyzed had detectable levels of glyphosate, the highest exposure recorded was 233 µg/L [40]. Mesnage et al. (2012) conducted a study on one farm family and found a maximum glyphosate concentration of 9.5 µg/L [39]. Curwin et al. (2007) conducted a study of farm and non-farm families and found that the non-farm family’s children had higher urinary glyphosate concentrations, which was assumed to be from residential use of glyphosate-based pesticide products [24]. Although urine samples were collected up to 5 days after pesticide application and glyphosate has a very short half-life [48], peak exposures may have been missed, which could partially be the reason for having no reported differences between the two groups. The study also observed a correlation between farmer urinary pesticide concentrations and spouse and children’s urinary concentrations [24,49], possibly due to para-occupational exposures among family members. Possible sources of para-occupational exposures were also identified in a glyphosate exposure study among amenity horticulturist workers [50]. Two HBM studies conducted among professional amenity horticulturists in Ireland reported maximum glyphosate concentrations of 7.36 µg/L and 10.66 µg/L [42,44]. Older studies (1991,1992) have been conducted among tree nursery and forest workers, but all the samples were non-detectable for glyphosate, probably due to the high analytical detection limits [45,46]. One recent study among 134 pesticide production workers, in four plants in Eastern China [47], found the highest urinary glyphosate concentrations ever reported for occupational exposures (median 292 µg/L, maximum 17.2 mg/L), that were considerably higher than in any other of the above listed occupational groups. 

Of the occupational studies that have evaluated glyphosate, only four have also measured AMPA. However, probably because of the higher LOQ/LOD in these studies, AMPA was not detected [39,46] or detection rates were low [43]. In the agricultural study by Perry et al. (2019), only one of the applicator samples that tested positive for glyphosate also tested positive for AMPA [43]. In contrast, Zhang et al. (2020) detected AMPA in the majority (81%) of pesticide production workers with a rather high median level of 68 µg/L (maximum 2.73 mg/L), but AMPA levels were only about a fifth the concentration of the glyphosate levels [47].

HBM studies with a focus on non-occupational exposure to glyphosate have been conducted in Germany [38,41,51], Ireland [52], Denmark [23], Colombia [53], Mexico [54], and the United States [15,36,55] and are summarized in Table 2. These studies include non-occupational exposures expected from dietary intake due to dietary exposures from pesticide food residues or contaminated water, or due to residential or bystander exposures from spraying events occurring in the surrounding area and due to para-occupational exposures, i.e., living with a pesticide user. A non-peer-reviewed environmental glyphosate exposure report (sponsored by Friends of the Earth Europe) across 18 European countries with 182 individuals detected glyphosate (LOQ 0.15 µg/L) in 44% of all samples. However, detection frequencies for each individual EU-state investigated ranged between 10% to 90%. The overall mean concentration was 0.21 µg/L, and the maximum concentration was 1.82 µg/L [35]. The German Environment Agency investigated the time trend of glyphosate exposures in the German population (2001–2015), using 24-h urine samples from the German Environmental Specimen Bank (ESB). Analyzed in the same laboratory as Hoppe (2013) [33], but now with an improved LOQ of 0.1 µg/L, they could detect and quantify glyphosate in 127 of the 399 urine samples (31.8%), with a maximum glyphosate level of 2.8 µg/L. Medians were below the LOQ for most years investigated, except for 2012 and 2013 (median 0.11 µg/L). The highest median was 0.18 µg/L for males in 2013 [38]. Another study conducted in Germany investigated 301 volunteers who participated in the cross-sectional Karlsruhe Metabolomics and Nutrition (KarMeN) study [41]. They reported 31% of the participants with concentrations above the LOQ of 0.2 µg/L for glyphosate with a maximum level of 1.36 µg/L. In 2017, a small exploratory HBM study conducted among the Irish population detected glyphosate levels in 10 (20%) out of 50 urine samples (LOQ 0.5 µg/L) with a maximum of 1.35 µg/L [52].

The Rancho Bernardo Study (RBS) of Healthy Aging in Southern California (from 1993 to 2016) collected 100 samples from residents older than 50 years of age (mean age of ~72 years old) and found mean glyphosate concentrations over the years investigated between 0.024 and 0.314 µg/L [55]. In the United States, Parvez et al. (2018) detected glyphosate in 90% of the urine samples collected from 71 pregnant women in Central Indiana USA, with a mean concentration of 3.40 µg/L and a maximum concentration of 7.20 µg/L [15]. These concentrations are substantially higher than any other environmental glyphosate exposure study. When comparing their results with other studies, their data would indicate to a hot-spot population, with even higher exposures than after occupational use. Unfortunately, no AMPA was measured to confirm their high glyphosate levels.

Of the 13 environmental studies that evaluated glyphosate (Table 2), only six also analyzed for AMPA [33,36,38,41,53,55]. In all of these studies, urinary AMPA levels were in the same concentration range as glyphosate. Hoppe (2013) [35] reported AMPA in 35.7% of samples with a mean of 0.18 µg/L (max 2.63 µg/L), very similar to the mean for glyphosate of 0.21 µg/L (max 1.82 µg/L) [33]. Conrad et al. (2017) detected AMPA in 40% of the 399 24-h samples collected (compared to 32% for glyphosate) with similar median concentrations for years where medians were available (e.g., median 2012 glyphosate: 0.11 µg/L and AMPA 0.12 µg/L) and similar maximum concentrations (1.88 µg/L for AMPA and 2.80 µg/L for glyphosate) [38]. They also reported that glyphosate and AMPA concentrations in urine were statistically correlated [38]. Such a correlation has also been reported in another study [36]. McGuire et al. (2016) found, among lactating women, no substantial difference between glyphosate and AMPA concentrations with 73% of samples above the LOQ and a mean concentration for AMPA of 0.30 µg/L, and a maximum of 1.33 µg/L for AMPA vs. 1.93 µg/L for glyphosate [36]. Similar results were found in the RBS Healthy Aging study in the US [55], with AMPA detected in 71% of the samples and a mean concentration of 0.285 µg/L comparable to the mean of glyphosate (0.314 µg/L). They also stated that the frequency of detection and concentration levels increased significantly over the time investigated (1993–2016) for both glyphosate and AMPA. In contrast, Varona et al. (2009) quantified AMPA in only 4% of their samples, making comparisons with glyphosate levels difficult [53]. Similarly, Soukup et al. (2020) only had 8% of their samples above the limit of quantification for glyphosate and AMPA [41]. 

Only four studies looked at mother and child exposures to glyphosate [23,24,39,40], with a further two looking at pregnant or lactating women [15,36]. In Knudsen et al. (2017) study of mother and children exposures had all detectable samples with a mean value of 1.28 µg/L and 1.96 µg/L for mothers and children, respectively [23]. Comparable values are reported for non-farm families in the US [24]. While McGuire et al. (2016) had a lower mean value of 0.28 µg/L for glyphosate among lactating women [36]. 

## 4. HBM Based Back-Calculated Oral Intake Equivalents 

Human biomonitoring (HBM) data reports concentrations of a chemical (or its metabolites) in human tissues (internal exposure; body burden) but requires further calculations to derive estimates of external exposure levels (e.g., daily intakes, external doses). Thus, the interpretation of the relationship between external doses and HBM data is highly dependent on the toxicokinetics of the substance and the knowledge thereof.

Toxicokinetic extrapolation from HBM data is used in the development of HBM guidance values (e.g., HBM-1), which are the equivalent for health-based guidance values (e.g., the acceptable daily intake (ADI)). A prerequisite for extrapolation is the availability of reliable toxicokinetic information, preferably from humans. To back-calculate urinary data to oral dose equivalents, the correct urinary excretion fractions are essential. Some studies refer to the urinary excretion fractions as the oral absorption rate. It is important to highlight that these two terms only have the same numerical value if all (100%) of the orally absorbed glyphosate is excreted as glyphosate in urine (without other excretion pathways, or without further metabolism or breakdown). There has been a number of studies that have presented methods for performing back calculations from HBM data to extrapolate for the external dose [56,57,58,59,60], which usually take into account a number of parameters such as the urine volume or creatinine level, the first order and total elimination kinetics, often expressed as the urinary excretion fraction, and the molecular weight of the substance/metabolite being analyzed in relation to its parent compound. The only study so far that had extrapolated urinary glyphosate levels to daily intakes and compared these with the European Food Safety Authority’s (EFSAs) ADI was conducted by Niemann et al. (2015) [32]. They applied a very simplified back-calculation approach with underlying assumptions on daily urine volume (2 L per day), body weight (60 kg), and oral absorption of glyphosate of 20%. To allow comparisons to this study, this paper conducted the same back-calculation approach but updated the assumption of oral absorption (based on animal data) with more recent data on human urinary excretion fractions. 

For glyphosate, until recently, the urinary excretion fractions were based on animal studies, and these fractions ranged from 19–34% of glyphosate being excreted in urine after oral exposure, and 20% was regarded as the most appropriate assumption [3,4,29,61]. Meanwhile, there have been more recent studies investigating humans instead of animals, i.e., dose recovery [30,31]. Two studies, investigating the total dose recovery for glyphosate in humans after oral glyphosate uptake reported very consistent urinary excretion fractions for glyphosate of 1–6% in three volunteers [31] and between 0.57 to 1.68% (mean 0.91%) in six male and six female volunteers [30]. These findings considerably deviate from the above previous assumptions. Table 3 shows the percentage of calculated glyphosate exposure compared to the ADI (0.5 mg/kg b.w./day) for non-occupationally exposed populations (from Table 2) using the back-calculation approach that Niemann et al. (2015) adopted [33], but using a 1% urinary excretion fraction instead of 20%. All of the environmental exposure studies had mean/median levels that were 2% of the ADI or less, and the maximum concentrations found in these studies were all less than 6%. One study on residential exposures showed median and maximum values that were 49% and 53% of the ADI, respectively, while another study reported a maximum value was 87% of EFSA’s ADI.

## 5. Discussion

For a substance that has been in use for such a long time and in such large quantities worldwide, there have been surprisingly few human biomonitoring (HBM) studies that measure glyphosate as an exposure biomarker in human biofluids, in either occupational or environmental settings. Even fewer studies are available that provide additional, quantitative and confirmatory AMPA data.

In this review, we identified 21 HBM studies that measured urinary glyphosate and/or AMPA across different populations (Table 1 and Table 2). A range of analytical methods employing different sensitivities has been used (LOD/LOQ ranging from 0.02 to 100 μg/L), impacting the reported frequency of detection (ranged from all non-detectable samples to 100% detection rate) and final data interpretation. Furthermore, across studies, the quantitative data was reported differently; several studies only presented detectable data, causing a positive bias. Other studies included non-detectable data by substituting those values with half the limit of detection or quantification, though the use of these substitution methods have been heavily debated when there is a high number of non-detectable data [62,63,64]. The US EPA advises this approach only if there are up to 15% non-detects [65]. Thus, if there are less than 50% of detectable samples (of all samples investigated), the median should be reported as below the LOQ/LOD (<LOQ/LOD). However, as heterogeneous as the data may appear, it shows that there is near omnipresent exposure to glyphosate (and AMPA) already in the general population that are environmentally exposed. Urinary glyphosate median levels are generally below 1 µg/L, and maximum levels are usually below 10 µg/L. Occupational exposures seem to add to the general body burden but with less than a 10 fold increase (maximum glyphosate concentration in a farmer’s urine 233 µg/L [40]). Occupational exposures among pesticide manufacturing workers are in orders of magnitude higher, with a reported mean urinary glyphosate concentration of 292 µg/L and a maximum concentration of 17.2 mg/L [47]. This suggests that exposures during glyphosate production and packaging could be substantial. The results found among this occupational group certainly warrant further investigation.

HBM data on glyphosate can not only be used to compare body burdens between different populations, identify highly exposed subpopulations, or follow time trends of exposure for statistical and epidemiological purposes, but it can also be used to perform back-calculations for exposures (such as daily intakes) and inform risk assessment and risk management procedures. In order to achieve this aim, interpretation of HBM data heavily relies on basic (human) toxicokinetic data and conversion/excretion fractions of exposure biomarkers (urinary glyphosate and AMPA in this case). Information on the rate and time period of absorption, elimination, and the calculated urinary excretion fractions of a compound allows internal concentrations to be back-calculated and to extrapolate for external exposures. 

The most significant impact on HBM based dose extrapolations for glyphosate have been two recent human metabolism studies by Faniband et al. (2020) [31] and Zoller et al. (2020) [30], both of which reported urinary excretion of ingested glyphosate as low as 1% instead of 20% (with the assumption that the remainder is mostly excreted unchanged via faeces [3]. Though based on only two studies and limited data, these studies are highly significant, given that there is a dearth of human data on urinary excretion fractions, and these studies produce similar results. In contrast, other studies derive these values from animal studies. One might interpret that glyphosate is 20 times less systemically available in humans than in rats with potential implications concerning its toxicity. However, when factoring this lower urinary excretion fraction into the back-calculation of oral intake, this suggests a 20-fold higher glyphosate intake for human populations than previously assumed based on urinary glyphosate data. With the previous 20% assumption, Niemann et al. (2015) [32] took the highest glyphosate concentration measured in the non-occupational studies and found it was 3.3 µg/kg b.w./day, a factor of 100 lower than the current EFSA ADI of 0.5 mg/kg b.w./day. Another review study calculated the systematic dose of glyphosate similar to Niemann et al. (2015) assuming a 20% absorption from the G.I. tract and complete excretion as glyphosate and found that exposures pose a *de minimis* risk [66]. Table 3’s calculations use a urinary excretion fraction of 1% and indicate that average glyphosate exposures in the non-occupational field are still well below the ADI, averaging out at only 0.2–2% of the EFSA ADI. However, upper-bound urinary glyphosate concentrations in several non-occupational populations indicate to intakes that could represent 5% [15] 6% [24], 53% [11], and 87% [53] of the ADI. This would considerably diminish the margin of safety assumed to be present for glyphosate exposure in the general population or potentially exposed subpopulations. Additionally, it should also be taken into consideration that environmental exposure concentrations of AMPA are similar to glyphosate, AMPA is reported as having a similar toxicological profile to glyphosate, as well as the glyphosate guidance values also applying to AMPA [3,67]. However, some considerations need to be taken into account when interpreting this data, including that the non-occupationally exposed populations were investigating environmental, residential, and para-occupational exposures. The highest values found were studies investigating aerial spraying in Columbia [11] and in a Sri Lankan agricultural district [53] (the non-agricultural region had much lower levels). Moreover, both studies had the inclusion of potentially occupationally exposed participants without indicating whether glyphosate was used before sample collection. Even when one takes into account the lower urinary excretion fraction, many of the non-occupationally exposed studies had upper-bound exposures less than 6% of the ADI. 

For occupational exposures, Niemann et al. (2015) [32] calculated a maximum systemic dose of 8.3 µg/kg b.w./day representing 8.3% of the EFSA acceptable operator exposure level (AOEL) of 0.1 mg/kg b.w./day. These extrapolations, however, did not include the later published exposure data in pesticide production plants by Zhang et al., (2020) [47] who showed exposures might be 100 times higher in some occupational settings than previously assumed. Following the assessment methods of Niemann et al. (2015) [32], using recent occupational data reported by Zhang et al., (2020) [47], the mean urinary glyphosate concentrations in workers in pesticide manufacturing would indicate a systemic dose of glyphosate that is approximately 10% of the AOEL, and maximum concentrations would indicate to 5 ½ times the AOEL limit. However, this was an occupational group engaged in the manufacturing of pesticides and other worker groups reported much lower exposure levels.

Zoller et al. (2020) also provides valuable first insights into human urinary excretion kinetics with peak concentrations occurring in urine approximately 5 h after oral uptake and an elimination half time of around 9 h [30]. Interestingly, their findings are rather similar to Connolly et al. (2019), who estimated the elimination half times in urine samples collected from seven amenity horticulture workers after spraying glyphosate to be between 5 ½ to 10 h [48]. Overall, these findings are lower than the urinary elimination kinetics known from animal studies, where the urinary half-life was calculated to be 72 h (50% elimination at 12 h) [68] and 48 h [3,69].

For AMPA, even less information is available on toxicity, human metabolism (internal and external), and exposure levels. From the environmental HBM studies (Table 2), we can deduce that urinary AMPA levels are within the same concentration range as glyphosate levels, even correlating with each other to some extend [36,38,41]. In the occupational studies listed in Table 1, urinary AMPA levels seem considerably lower in relation to urinary glyphosate levels. This might indicate that urinary AMPA is predominately caused by direct AMPA uptake via food residues and water, and only to a lesser extent by human metabolism of glyphosate. In confirmation, animal studies show that only a very small amount of glyphosate may be metabolized to AMPA (0.2–0.3%), possibly by gut microflora, and excreted in urine [3,4,29,61]. In Zoller et al. (2020), the human volunteers were simultaneously exposed to glyphosate and AMPA consuming contaminated falafel. However, the ingested glyphosate amount (~200 µg) was approximately 100 times larger than the ingested AMPA amount (~1.6 µg). They concluded that 23% of the AMPA dose was excreted in urine as AMPA. Assuming that no metabolism of glyphosate to AMPA occurred, only 0.3% of all of the urinary AMPA originated from the glyphosate [30]. Thus, the “real” urinary excretion fraction of AMPA (originating from oral AMPA) is certainly below 23% but can only be robustly determined after dosage of pure AMPA, not accompanied by glyphosate.

Irrespective of the final quantity of this urinary excretion fraction of AMPA from glyphosate exposure (or from AMPA exposure), it can be assumed that the majority of the urinary AMPA observed in environmental HBM studies stems from exposure to AMPA and not from exposure to glyphosate. Thus, analyzing both AMPA and glyphosate in urine is important to elucidate reasons for some differences in internal concentrations of glyphosate and AMPA potentially caused by different exposure sources and pathways [38,70], which is in agreement with the opinion of the WHO [71] that glyphosate together with AMPA and some other degradation products should be regarded as residues of toxicological concern. In 2011, the JMPR established a group ADI of 0–1 mg/kg bw for the sum of glyphosate, AMPA (and N-acetyl glyphosate and N-acetyl-AMPA). Thus, the inclusion of AMPA in HBM-based exposure assessments are warranted to further increase the combined knowledge of environmentally caused glyphosate/AMPA body burdens. This study compared reported non-occupational glyphosate exposures with the ADI, but solely focused on glyphosate (Table 3). Considerations should be made for the potential of additional exposures to AMPA. 

## 6. Conclusions

Glyphosate is the highest volume used herbicide in the world and has been found to be ubiquitous in the environment. Nevertheless, there is a dearth of information about glyphosate exposures and very few HBM studies for glyphosate, as well as its main degradation product aminomethylphosphonic acid (AMPA) exist. Additionally, further data is required to understand the role of AMPA in relation to glyphosate exposures and the human toxicokinetics of both glyphosate and AMPA.

Consequently, glyphosate and AMPA were selected as priority substances in the Human Biomonitoring for Europe initiative (HBME4U) [71]. HBM4EU is a European Joint Program that involves 30 countries, the European Environment Agency and the European Commission, co-funded under Horizon 2020 and has been running from 2017–2021. The European Environment Agency and the European Commission have the goal to harmonize, coordinate, and advance HBM in Europe, connecting HBM research with policy to address societal concerns [72,73,74]. Thus, several issues with glyphosate/AMPA HBM need to be tackled, as described in this paper. Analytical methodologies applied to quantify environmental exposures need to reach sufficient sensitivities (LOQ of 0.1 µg/L or better) and stringent quality assessment schemes need to be established to ensure accuracy, but also the comparability of the analytical data for both glyphosate and AMPA. Moreover, to ensure comparability of the HBM data, sampling strategies and target populations need to be harmonized, and exposed subpopulations need to be identified (e.g., children, para-occupational exposures, occupational exposures). To facilitate direct interpretation of the HBM data, HBM based guidance values should be established [75], similar to the biomonitoring equivalents (BE) [76] and HBM values (HBM I, II) [56], which directly relates to urinary concentrations already established by ADI values. New, human-based toxicokinetic data by Faniband et al. (2020) [31] and Zoller et al. (2020) [30] might enable the derivation of such values and directly allow the deduction of urinary concentrations if acceptable/tolerable intakes are exceeded by environmental and/or occupational exposure [73,74]. Such information can robustly advise governing bodies or, more specifically, the current approval of glyphosate expiring in Europe in December 2022 [77].

## Figures and Tables

**Table 1:** Glyphosate (GLY) occupational exposure assessment studies employing biological monitoring methods via urine sampling ^1^.

Ref:	Country	Type of Study	Sampling Population	Urine Sample Type	Analytical Method	*GLY Conc.* (µg/L)				*AMPA Conc.* (µg/L)			
						LOD/LOQ	% above LOQ/LOD	Average	Max	LOD/LOQ	% above LOQ/LOD	Average	Max
Zhang, F. et al., 2020 [47]	China	Pesticide production plants	Workers across 4 production plants	End of work shift samples. *n* = 134	GC-MS ^2^	LOD 20	~87%	Mean 292	17,200	10	~81%	Median 68	2730
Perry, M.J. et al., 2019 [43] ^3^	US	US agricultural cohort study	18 farmers—8 hrs after application and 17 non-applicators	Spot urine samples ^4^	LC-MS/MS ^5^	LOD 0.4	39%	Median < LOD	12.0	1	6%	Median < LOD	NR ^6^
Connolly, A. et al., 2018 [42]	Ireland	Horticulture amenity gardening	20 workers for 29 tasks. A total of 125 individual samples	Individual full void samples ^7^	LC-MS/MS	LOQ 0.5	93%	Peak values GM 1.90 AM 2.53	7.36	N/A	N/A	N/A	N/A
Connolly, A. et al., 2017 [44]	Ireland	Horticulture amenity gardening	17 workers—31 paired samples	Spot samples ^8^	LC-MS/MS	LOQ 0.5	55%	GM 0.66 AM 1.35	10.66	N/A	N/A	N/A	N/A
Mesnage, R. et al., 2012 [39]	France	Farm family exposure study	1 farmer, spouse and 3 children	24-h urine over three days	HPLC-ESI-MS ^9^	LOD 1 LOQ 2	NR^2^	Overall results not given	9.5	NR	0	Non detect	Non detect
Curwin, B. et al., 2007 [24] ^10^	USA—Iowa	Farm and ‘non-farm’ familiesinvestigating take-home pesticide exposure	**Farm** Father (*n* = 24) Mother (*n* = 24) Child (*n* = 25)	Two full void spot urine samples. ^11^	Immunoassay (fluorescent microbeads)	LOD 0.9	Overall ~77%	GM **Farm**: Father 1.9 Mother 1.5 Child 2.0	18	N/A	N/A	N/A	N/A
Acquavella, J.F. et al., 2004 [40] ^12^	USA—South Carolina, Minnesota	Occupational and residential exposures in an agricultural setting	48 farmers, 48 spouses and 79 children	24-hr composite urine samples ^13^	HPLC following ion exchange	LOD 1	Farmer 60% Spouse 4% Child 12%	Farmer GM 3.2	Farmer 233 Spouse 3 Child 29	N/A	N/A	N/A	N/A
Lavy, T.L et al., 1992 [45]	United States	Conifer Seedling Nursery	14 workers	24-h urine ^14^	Not specified	LOQ 10	0	0	0	N/A	N/A	N/A	N/A
Jauhiainen, A. et al., 1991 [46]	Finland 1988	Forest workers	5 Forest workers and 5 control group	Post work shift samples ^15^	GC with a 63Ni-electron capture detector	LOD 100	0	N/A	<LOD ^16^	LOD 50	0	N/A	N/A

^1^ N/A: Information not available due to single measurements or not given in the literature. ^2^ Gas chromatography-mass spectrometry. ^3^ Only reporting the results of the occupationally exposed group (e.g., 18 farmers). ^4^ From 1997 and 1998, long-term cryopreservation. ^5^ Liquid chromatography tandem mass spectrometry. ^6^ Not reported. ^7^ Multiple individual full void samples (before and after the work task and the following morning void), the original paper also provided creatinine adjusted results. ^8^ Samples were taken before and after the work tasks, the original paper also provided creatinine adjusted results. ^9^ High-performance liquid chromatography, electrospray ionization, and mass spectrometry. ^10^ The results of the non-farm family are available in Table 2 under environmental exposures. ^11^ Two visits involved the collection of two full void spot urine samples, one evening and the following morning void. Results creatinine adjusted. ^12^ The results reported in this table are the concentrations found on the day of pesticide application, with the overall highest concentrations. ^13^ The 24-h composite urine samples were collected the day before spraying, the day of spraying and the following three days after the spraying event. ^14^ Samples were collected from each worker each day for a minimum of 8 weeks, to calculate seasonal exposures. ^15^ Urine sample was taken at the end of the workday during the spraying week and also after a 3 week period. ^16^ One urine sample was further quantified and had 85 μg/L of glyphosate.

**Table 2:** Glyphosate (GLY) non-occupational exposure assessment studies employing biological monitoring methods via urine sampling from most recent publications ^1^.

Ref:	Country	Type of Study	Sampling Population	Urine Sample Type	Analytical Method	*GLY Conc.* (µg/L)				*AMPA Conc.* (µg/L)			
						LOD/LOQ	% above LOQ/LOD	Average	Max	LOD/LOQ	% above LOQ/LOD	Average	Max
Soukup, S.T. et al., 2020 [41]	Germany	KarMeN study	Cross-sectional study of 301 adults	24 h full void samples	LC-MS/MS	LOD/LOQ 0.05/0.20	8% ≥ LOQ ^2^	Median < LOQ	1.37	LOD/LOQ 0.09/0.20	8% ≥ LOQ	Median < LOQ	1.53
Connolly, A. et al., 2018 [52]	Ireland	Pilot study	50 adults	One spot sample	LC-MS/MS	LOQ 0.5	20%	Median < LOQ	1.35	N/A	N/A	N/A	N/A
Parvez, S. et al., 2018 [15]	USA—Indiana	Environmental study	71 pregnant women	NR	LC-MS/MS	LOQ 0.5 LOD 0.1	93% > LOD	Mean 3.40	7.20	N/A	N/A	N/A	N/A
Conrad, A. et al., 2017 [38]	Germany	General population	399 samples (20–29 years old)	24-h urine samples ^3^	GC-MS/MS ^4^	LOQ 0.1	~32%	Median < LOQ	2.80	LOQ 0.1	~40%	Median < LOQ	1.88
Knudsen, L.E. et al., 2017 [23]	Denmark	Mother and child study	Mother (*n* = 13) Children 6–11 (*n* = 14)	Spot samples ^5^	ELISA ^6^	LOD 0.0751	100%	Mean Mothers 1.28 Children 1.96	3.31	N/A	N/A	N/A	N/A
Mills, P.J. et al., 2017 [55] ^7^	USA—California	Older Adults between 1993 and 2016	Adults more than 50 years old	Morning spot urine samples (*n* = 100)	HPLC-MS ^8^	LOD 0.03	70%	Mean 0.314	NR ^9^	LOD 0.04	71%	0.285	NR
Rendon-von Osten, L. and Dzul-Caamal, R. 2017	Mexico	Farmers and Fishermen ^10^	Men between 30–50 years old	Morning spot urine samples	ELISA	LOQ 1.0	NR	Mean of 1 group (*n* = 15) 0.47	NR	N/A	N/A	N/A	N/A
McGuire, M.K. et al., 2016 [36]	USA—Washington and Idaho	Lactating women	Analysing glyphosate in milk and urine	Midstream urine spot sample (*n* = 40)	LC-MS/MS	LOD/LOQ 0.02/0.10	~93% > LOD	Mean 0.28	1.93	LOD/LOQ 0.03/0.10	95% > LOD	0.30	1.33
Jayasumana, C. et al., 2015 [11] ^11^	Sri Lanka	Investigate Sri Lankan Agricultural Nephropathy (SAN) patients	Patients with SAN, healthy group from the area and a different area	Morning spot urine samples. 10 samples per group	ELISA validation compared with GC-MS	LOD 0.6	100%	Medians SAN endemic area 73.5 Non endemic area 3.3	Peak ≥ 80	N/A	N/A	N/A	N/A
Krüger, M. et al., 2014 [51]	Europe	Human and animal exposure study	conventional diet *n* = 99 and organic diet *n* = 41. Healthy *n* = 102 and chronically diseased *n* = 199	NR	ELISA partly validated against GC-MS	LOD/LOQ unknown	NR	NR	~5	N/A	N/A	N/A	N/A
Hoppe, H.W. 2013 [33] ^12^	Europe	Environmental exposures	18 EU countries	Not specified, urine samples n = 182	GC-MSMS	LOQ 0.15	44%	Median < LOQ	1.82	LOQ 0.15	36%	Median < LOQ	2.6
Varona, M. et al., 2009 [53] ^13^	Columbia	Aerial spraying	106 samples analysed ^14^	Spot urine samples	G.C. with electron micro-capture detector	LOD 0.5 LOQ 2	~40%	Median < LOQ	130	LOD 1.0 LOQ 15	3.8%	Median < LOQ	56
Curwin, B. et al., 2007 [23] ^15^	USA—Iowa	Farm and ‘non-farm’ families investigating take-home pesticide exposure	Non-farm Father (*n* = 23) Mother (*n* = 24) Child (*n* = 25)	Two full void spot urine samples ^16^	Immunoassay (fluorescent microbeads)	LOD 0.9	77%	GM Non-farm Father 1.4 Mother 1.2 Child 2.7	9.4	N/A	N/A	N/A	N/A

^1^ N/A: Not Available: Information not available due to single measurements or not given in the literature. ^2^ Stated as glyphosate and/or AMPA levels that were above the limit of quantitation, do not specify the % of each. ^3^ German ESB—cryo-preserved urine samples. ^4^ Gas chromatography tandem-mass spectrometry. ^5^ Consortium to Perform Human biomonitoring on European Scale (COPHES), a pilot study the Demonstration of a study to Coordinate and Perform Human biomonitoring on a European Scale (DEMOCOPHES). ^6^ Enzyme-linked immunosorbent assay (ELISA). ^7^ The results reported are only for the years 2014-2016. The paper reports for four other time periods, the 2014–2016 time period was the only one that found more than 50% of detectable glyphosate levels in the samples. ^8^ High-performance liquid chromatography coupled with mass spectrometry. ^9^ Not reported. ^10^ A cross-section prospective study from subsistence farmers in five communities. ^11^ The Jayasumana et al. 2015 analyses samples from three groups, 10 people per group. The results shown here are for the two control groups, the healthy group from the Sri Lankan Agricultural Nephropathy (SAN) area and the group located in a different area without a high rate of SAN. ^12^ Not a peer reviewed study. ^13^ The Varona M. et al. 2009 article is in Spanish, with only the abstract translated to English. ^14^ This study was of 112 people living in an area within 5 days of aerial spraying. 106 samples analysed. Although this study was investigating exposures from aerial spraying, the study did include occupationally exposure participants; however, the study did not indicate whether pesticide use occurred before sample collection. ^15^ This results of the farm family are available in Table 1 under occupational exposures. ^16^ Two visits involved the collection of two full void spot urine samples, one evening (within 5 days of a spraying event) and the following morning void. Results creatinine adjusted.

**Table 3:** The glyphosate concentrations in human urine samples (average and maximum values) from the non-occupationally exposed studies (Table 2) compared to the EFSA ADI ^1^ using a 1% urinary excretion fraction ^2^.

Study	Percentage of EFSA ADI [%]	
	Average	Max
Soukup et al., 2020 [41]	<LOQ (median)	1%
Connolly et al., 2018 [52]	<LOQ (median)	1%
Parvez et al., 2018 [14]	2% (mean)	5%
Conrad et al., 2017 [38]	<LOQ (median)	2%
Knudsen et al., 2017 [23]	1% (mothers, mean) 1% (children, mean)	2%
Mills et al., 2017 [55]	0.2% (mean)	NR ^3^
Rendon-von Osten. et al., 2017 [54]	0.3% (mean)	NR
McGuire et al., 2016 [36]	0.2% (mean)	1%
Jayasumana et al., 2015 [11]		53% ^4^
(control populations)		
SAN endemic areas	49% (median)	
non-endemic area	6% (median)	
Krüger et al., 2014 [51]	NR	3%
Hoppe, 2013 [33]	<LOQ (median)	1%
Varona et al., 2009 [53]	<LOQ (median)	87% ^4^
Curwin et al., 2007 [24] (Non-farm family)	1% (father, GM) 1% (mother, GM) 2% (child, GM)	6%

^1^ The European Food Safety Authority (EFSA) acceptable daily intake (ADI) allowance is expressed as the amount mass of glyphosate, per kilogram of body weight per day (mg/kg b.w./day). ^2^ AMPA have similar concentrations in environmentally exposed individuals (results not presented). AMPA has been stated to have a similar toxicological profile to glyphosate, and glyphosate reference values also apply to AMPA (EFSA, 2015). ^3^ Not reported. ^4^ These studies were investigating non-occupational exposures; however, both reported the inclusion of potentially occupationally exposed participants. Therefore, we cannot eliminate the possibility that the upper exposure levels might be influenced by occupational exposure sources.
